# To the Medical Profession of the United States

**Published:** 1871-12

**Authors:** 


### To the Medical Profession of the United States.

---

The terrible calamity which has recently fallen upon the city of Chicago and upon various portions of the Northwest, has awakened the sympathy of the world, and both money and material have been sent to the suffering districts with an abundance and alacrity which has never before been witnessed; but no one who has considered the extent of the losses, or the amount of suffering entailed, can feel any apprehension that the work of charity is likely to be overdone, nor, indeed, that it will be possible to fill the measure of the actual want. There ought to be no relaxation in these general measures of relief for many months to come, nor is it probable there will be; but there is one class whose misfortunes appeal most especially to the members of our profession. More than a hundred physicians in Chicago, and probably as many more in other parts of the Northwest, have lost all they possessed. The intelligence received by us, from trustworthy sources, is of the most painful character; and it is with reluctance that we make public the fact that up to this moment seventy-seven physicians in Chicago alone have been driven to the necessity of placing their names upon the list of those requiring pecuniary aid. God forbid that we should delay to give them help. We beg you to reflect that the situation of these physicians, with their families, is peculiar. The laborer may find employment at his usual wages; the merchant may buy and build upon his credit; the clergyman has his congregation, much less able to pay than formerly, but nevertheless responsible for his support. The physician has ordinarily none of these resources. With neither house nor furniture, horse, carriage, nor instruments, he must do what little he can, and wait the slow returns from a population reduced, like himself, almost or quite to beggary. Our calling is never a lucerative one, but in Chicago to-day it can hardly be expected to supply the necessities of life, and perhaps not for a year to come.

In our opinion these seventy-seven doctors, and probably as many more, will need from $500 to $1,000 each to carry them safely through the year, and to put them once more upon their feet. From $50,000 to $80,000 is our lowest estimate of what should be sent to the Chicago doctors' relief fund; with this money they may be placed upon salaries, and in return perform such public services among the sick and poor as may be required.

We have already received from the physicians of New York over $5,000, of which sum $4,000 has been sent to Chicago. The remainder is retained for the purpose of aiding the physicians of Wisconsin and Michigan, and will be forwarded to Chicago or elsewhere as soon as we receive information as to where it is especially needed. Many of the other large cities have sent in similar contributions. We have no means of knowing how much has been contributed, but we have no doubt the sum is totally inadequate to meet the exigencies of the case. It is proposed, therefore, to continue the organization of the committee, and not to cease efforts during the winter, unless its services should seem to be no longer required.

To physicians living in scattered districts we take the liberty of suggesting organized action through country or local associations. Those who prefer can send their contributions direct to Walter Hay, M. D., Secretary of the Chicago Relief Committee, No. 384 Michigan Avenue, Chicago; or to the Secretary of this Committee, Samuel T. Hubbard, M. D., No. 27 West Ninth Street, New York. We earnestly hope that no physician in the United States will omit to contribute something, however small the amount may be, to this charity—in any way and through any channel they choose—but that they all give, and that speedily. If our medical brethren knew only a few of the examples of individual suffering which have come to our knowledge, but which we do not feel at liberty to publish, their contributions would not be delayed.

Surgical instruments may be sent to the following surgical instrument-makers in this city, by whom they will be forwarded free of charge: George Tieman & Co., 67 Chatham Street; Darrow & Co., 1227 Broadway; Otto & Reynders, 64 Chatham Street; Shepard & Dudley (formerly Ford & Co.,) 150 William Street; Stohlman, Pfarre & Co., 107 East Twenty-eight Street.

Books will be received and forwarded by Wm. Wood & Co., No. 27 Great Jones Street.

It may be necessary to add in explanation of the funds originally intended only for the physicians of Chicago, that the probability of an appeal from the burnt districts of Michigan and Wisconsin, determined the Committee to reserve a small portion for such an exigency, and further, that in consideration of the fact the medical students of this city have given very liberally to this fund, it was determined to suggest to those having in charge the distribution of these charities, that they will not overlook the claims of medical students who may in the same manner have been left destitute.

Frank H. Hamilton, M. D., *Chairman.*
Alfred E. Purdy, M. D., *Secretary.*
Samuel T. Hubbard, M. D., *Treasurer.*
Geo. F. Shrady, M. D.,
Chas. McMillan, M. D.,
Ed. S. Dunster, M. D.,
A. Underhill, M. D.,
J. C. Peters, M. D.,
F. A. Castle, M. D.,
F. A. Burrall, M. D.

